# Potentially traumatic events have negative and positive effects on loneliness, depending on PTSD-symptom levels: evidence from a population-based prospective comparative study

**DOI:** 10.1007/s00127-017-1476-8

**Published:** 2017-12-29

**Authors:** Peter G. van der Velden, Bas Pijnappel, Erik van der Meulen

**Affiliations:** 1 INTERVICT, Tilburg University, PO Box 90153, 5000 LE Tilburg, The Netherlands; 2 Jacob-Roelandslyceum, Boxtel, The Netherlands

**Keywords:** Loneliness, Trauma, PTSD, Mental health, Prospective study

## Abstract

**Purpose:**

Examine to what extent adults affected by recent potentially traumatic events (PTE) with different PTSD-symptom levels are more at risk for post-event loneliness than non-affected adults are in the same study period.

**Methods:**

We extracted data from the Dutch longitudinal LISS panel to measure pre-event loneliness (2011) and post-event loneliness (2013 and 2014), pre-event mental health problems (2011), PTE and PTSD symptoms (2012). This panel is based on a traditional random sample drawn from the population register by Statistics Netherlands.

**Results:**

Results of the multinomial logistic regression analyses showed that affected adults with high levels of PTSD symptoms were more at risk for high levels of post-event loneliness than affected adults with very low PTSD-symptom levels and non-affected adults, while controlling for pre-event loneliness, pre-event mental health problems and demographics. However, affected adults with very low levels of PTSD symptoms compared to non-affected adults were less at risk for medium and high levels of post-event loneliness while controlling for the same variables. Yet, pre-event loneliness appeared to be the strongest independent predictor of loneliness at later stages: more than 80% with high pre-event levels had high post-event levels at both follow-ups.

**Conclusions:**

Remarkably, potentially traumatic events have depending on PTSD-symptom levels both negative and positive effects on post-event loneliness in favor of affected adults with very low PTSD symptoms levels. However, post-event levels at later stages are predominantly determined by pre-event loneliness levels.

## Introduction

The adverse effects of potentially traumatic events on (mental) health such as (traffic) accidents, (intimate partner) violence, life-threatening diseases, (sudden) death of a significant other and disasters are well-documented nowadays. Affected adults may suffer from various post-event stress symptoms such as fear and anxiety, grief and sadness, frustrations and anger, pain, cardiovascular problems, sleep problems and fatigue in the short, medium and long term. A variable minority suffer from mental disorders such as acute stress disorder, major depression and posttraumatic stress disorder [1–8]. However, the consequences of potentially traumatic events (PTE) are not restricted to (severe) (mental) health problems. PTE may affect other important resource in life such as safety and justice, work and income, social networks and relationships that may cause stress and negatively affect well-being. Importantly, each year a significant minority of the adults is confronted with PTE [9, 10].

People affected by PTE strive to restore important resources lost [11, 12]. To a certain degree, they possess the capability to manage one’s personal functioning in the myriad of environmental demands of the aftermath of PTE, also depending on the consequences of the PTE. Coping self-efficacy levels of the affected may help to reduce sources of stress and stress symptoms [13–15]. The presence of help and support from family, friends, colleagues, peers as well as involved professionals may further help to reduce both sources of stress as well as stress symptoms. Higher levels of perceived social support, and especially emotional support, are associated with lower levels of mental health problems in general and post-event mental health problems such as PTSD-symptomatology [16–19]. However, on the long term (persistent) problems and how people cope with them may reduce support [18, 20–22].

Therefore, loneliness will likely hinder post-event recovery efforts. Post-event experiences of unpleasant or inadmissible lack of (quality of) certain relationships [23] could increase the risk for ongoing severe posttraumatic stress symptoms. Non-trauma studies in the past decades have shown that loneliness, i.e. the subjective feeling referring to the perceived inadequacy of one’s social relationships [23–28], is clearly negatively associated with health. In a recent overview of 40 systematic reviews and meta-analysis of studies on the health consequences of loneliness, Leigh-Hunt et al. [29] concluded that there is consistent evidence linking loneliness to increased all-cause mortality, worse mental health and negative cardiovascular outcomes.

An important question in this perspective is to what extent potentially traumatic events (PTE) and PTE-related symptoms contribute to post-trauma loneliness. Insight in these factors may help to develop and target post-event mental health policies and interventions, to reduce or prevent loneliness. To date, many correlations of loneliness have been identified varying from employment to low self-esteem [25, 26, 28], but a limited number of studies focussed on PTE and related symptoms as predictors of loneliness.

For example, Hawthorne [30] assessed the association between lifetime trauma exposure and social isolation, a concept strongly related to loneliness [31]. In this cross-sectional study, Australian adults with three or more lifetime trauma exposures were more at risk for current social isolation than those with two or less. However, when current depression disorders were added to the list of eight predictors of social isolation, lifetime exposure was not an independent predictor. The results of the cross-sectional study of Palgi et al. [32] partly differ from the study of Hawthorne [30]. They showed that lifetime potential traumatic events among older US adults were positively related to loneliness. Adults with two or more lifetime events were more at risk for loneliness than adults without these experiences, while current depressive symptoms were taken into account too. In a cross-sectional study among older US veterans of Kuwert et al. [33], the number of lifetime traumatic events, current PTSD and depression symptoms were significantly associated with current loneliness over and above other variables, such as demographics and support. The cross-sectional study of Lasgaard et al. [34] demonstrated that having a life-threatening somatic condition was an independent risk factor for both moderate and severe loneliness over and above a list of demographic, work- and health-related variables. However, recalled lifetime trauma exposure or events may be sensitive to recall bias, influencing the strength of the associations between both [35, 36]. One of the exceptions, among others, is becoming a widow. The possible effects of being a widow was assessed in the large cross-sectional study of Victor et al. [37] among older people in Great Britain. It showed that widowed compared to married persons were more at risk for loneliness, over and above for (recalled) changes in loneliness in past 10 years, health and other variables.

Solomon et al. [38] conducted a three-wave post-war study among Israeli veterans covering a period of 20 years. Findings showed that loneliness levels were higher at all waves among veterans with combat stress reaction (CSR; succumb to stress and suffer a psychic breakdown on the battlefield) compared to soldiers without CSR. Baseline PTSD symptom levels did not predict future loneliness levels, although they were significantly related on a cross-sectional level. The two-wave study of Cohen-Mansfield et al. [39] among older persons in Israel showed that the number of lifetime traumatic events were independently associated with loneliness at the first wave, especially among married persons, over and above demographics, health variables and social network variables. However, lifetime traumatic events were not associated with becoming lonely in the period of 3.5 years between both waves in contrast to poorer health.

These findings suggest that potentially traumatic events as well as post-event posttraumatic stress symptoms, besides pre-event mental health, may increase the risk for post-event loneliness in the short and medium term. However, it is unclear to what extent these variables remain significant predictors of post-event loneliness when pre-event loneliness is taken into account. For instance, in the longitudinal study of Cacioppo et al. [40], loneliness among older people in the US was relatively stable over a period of 3 years, and loneliness at baseline appeared to be a significant, strong and dominant independent predictor of loneliness at follow-ups compared to, although also significant predictors, social support and depression symptoms at baseline. In addition, in their systematic review of 54 prospective longitudinal studies of PTSD, DiGangi et al. [41] came to the worrying conclusion that many variables, previously considered outcomes of trauma, are pre-trauma risk factors. These findings underscore the necessity of, when studying the effects of trauma on loneliness, prospective longitudinal study designs with pre-trauma measures on loneliness. However, to the best of our knowledge, to date such studies are absent.

Aim of the present study is assess to what extent potentially traumatic events and posttraumatic stress predict post-trauma loneliness using such a rigorous prospective study design, with pre-event and post-event measures of loneliness and a comparison group of non-affected adults. Main research questions are: (a) to what extent are adults affected by a PTE and subgroups with low to high levels of posttraumatic stress symptom more at risk for loneliness at later stages than non-affected adults? And (b) do affected adults with low levels of posttraumatic stress symptom differ from affected adults with higher levels of symptoms in post-event loneliness?

Based on the outcomes of previous studies on predictors of loneliness, we controlled for the possible effects of pre-event mental health, pre-event loneliness, age, gender, having a partner, and education level. We focus on relatively recent acute potentially traumatic events that assessed the predictive values for loneliness at two follow-ups with a 1-year interval.

## Methods

### Procedures and participants

CentERdata (Tilburg, the Netherlands) operates the Longitudinal Internet Studies for the Social Sciences-panel (LISS-panel) starting in 2007 [42]. This panel, based on a traditional random sample drawn from the population register by Statistics Netherlands, consists of about 7000 individuals with a yearly attrition rate of about 10%. They are frequently invited to complete online surveys (more information about the LISS-panel and how to gain free access can be found in English at: http://www.lissdata.nl). There are several so-called core studies that are repeated each year in the same period (with a few exceptions), such as the core studies on health that are conducted each year November–December.

Proposals for studies, such as the studies on trauma we included in the present study (see below), are first evaluated by the Board of Overseers, the Internal Review Board. During the recruitment of the panel, respondents who agreed to participate in the panel received a confirmation e-mail, and a letter with login code. With the login code provided, they could confirm their willingness to participate and immediately start the first interview (i.e. double consent was ensured, for details see [43]).

Using this panel, in 2012 three surveys on trauma were conducted (T2a, April^response^ = 78.4%; T2b, August^response^ = 83.5%, T2c, December^response^ = 86.3%) [14, 43]. In total, 4857 respondents participated in all three surveys on trauma and their data was extracted for this study. We also extracted data from studies on Social Integration and Leisure to obtain data on pre- and post-event loneliness, that were conducted in the spring of 2011 (T1a, February–March^response^ = 63.1%), 2013 (T3, February–March^response^ = 86.0%), and of 2014 (T4, February–March^response^ = 86.0%). To obtain data on pre-event mental health problems, we extracted data from a survey on health conducted at the end of 2011 (T1b, November–December 2011, response = 77.2%). In total, 3244 respondents participated in these seven surveys.

We next made a distinction between by PTE’s affected respondents and comparison respondents not confronted with PTE’s or other life events in the 2 years before T2a and not between T2a and T2c (N^no PTE^ = 1513; questions on PTE see below). With respect to the affected respondents, we also excluded respondents confronted with PTE’s in the 2 years before T2a to ascertain that they (also) were not confronted with PTE’s about a year before T1, or between T1 and T2a. We finally selected respondents confronted with a PTE between T2a and T2b, and/or between T2b and T2c (*n*^total PTE^ = 285).

### Variables

#### Loneliness

Pre- and post-event loneliness was assessed at T1a, T3 and T4 using the six-item De Jong Gierveld Loneliness Scale [44]. Respondents are asked to rate items such as ‘I often feel deserted’ and *‘*there are enough people I can count on in case of a misfortune’ on three-point Likert scales (1 = yes, 2 = more or less, 3 = no). For the present study, we calculated the total score after recoding the three negative formulated items (0.76 ≤ Cronbach’s Alpha’s ≤ 0.81 across 3 loneliness assessments). For the present study, we calculated the total score after recoding the three negative formulated items, but we did not use the prior dichotomization of the answer categories [45]. The reason was that the total score scale of loneliness without prior dichotomization enabled the categorization of sub groups with different levels of loneliness with almost equal cell counts somewhat better [Spearman’s rank (rho) correlation between scales with and without prior dichotomization is 0.99]. According to the cut-offs of the manual^1^ about 10% (*n* = 176) had severe loneliness scores.

#### Mental health problems

Pre-event mental health problems were examined at T1b using the Mental Health Index or Inventory (5-item sub scale of the MOS 36-item short-form health survey) [46, 47]. Respondents were asked to rate their mental health during the past month on six-point Likert scales, such as ‘This past month I felt very anxious’ and ‘I felt depressed and gloomy’ (1 = never, 2 = seldom, 3 = sometimes, 4 = often, 5 = mostly, 6 = continuously). After recoding the third and fifth item, the total scores were computed (Cronbach’s Alpha = 0.84).

#### Potentially traumatic events

Confrontations with potentially traumatic events were examined at T2a, T2b and T2c. Respondents were administered a list of ten potentially traumatic events [i.e.: serious threat(s); physical violence; robbery; traffic accident; airplane incident; fire; burglary; serious infection/contamination such as HIV, legionella, poison; sexual violence/abuse; death of significant other/colleague] and an open question with regard to having experienced another potentially traumatic event in the past 2 years not listed that were recoded afterwards [14, 43]. In case respondents were confronted with more than one event in the past 2 years, they were asked to focus on the most drastic event (1 = 1 week ago, 2 = 2 weeks ago, 3 = 3 weeks ago, 4 = 4 weeks ago, 5 = 1–2 months ago, 6 = 3–4 months ago, 7 = 5–6 months ago, 8 = 7–12 months ago, 9 = more than 1 year ago). In case respondents reported life events that cannot be considered PTE (such as conflicts in the family, job loss) and were not confronted with PTE, they were excluded from the analyses.

#### Posttraumatic stress symptoms

To assess PTSD-symptom severity (DSM-IV), the 15-item impact of event scale (IES) [48, 49] assessing intrusion and avoidance reactions during the past week was administered at T2c. In addition, the seven hyper arousal items from the IES-R [50] were added while the original scoring system of the IES was retained. This approach has been used in previous research [15] and has the benefit of comparability with results obtained using the original IES, while still allowing for the measurement of all three symptom clusters (avoidance, re-experiencing, hyper arousal) of PTSD (DSM-IV). Factor analysis confirmed the general three-factor structure [15]. Affected respondents were asked, while taking (the most recent) potential traumatic event in mind, to rate items such as: during the past week ‘I thought about it when I did not mean to’ or ‘I stayed away from reminders of it’ and ‘I had trouble concentrating’ on four-point Likert sales (0 = not at all, 1 = rarely, 3 = sometimes, 5 = often). We call this instrument the IESplus (Cronbach’s Alpha = 0.94).

**Fig. 1:**
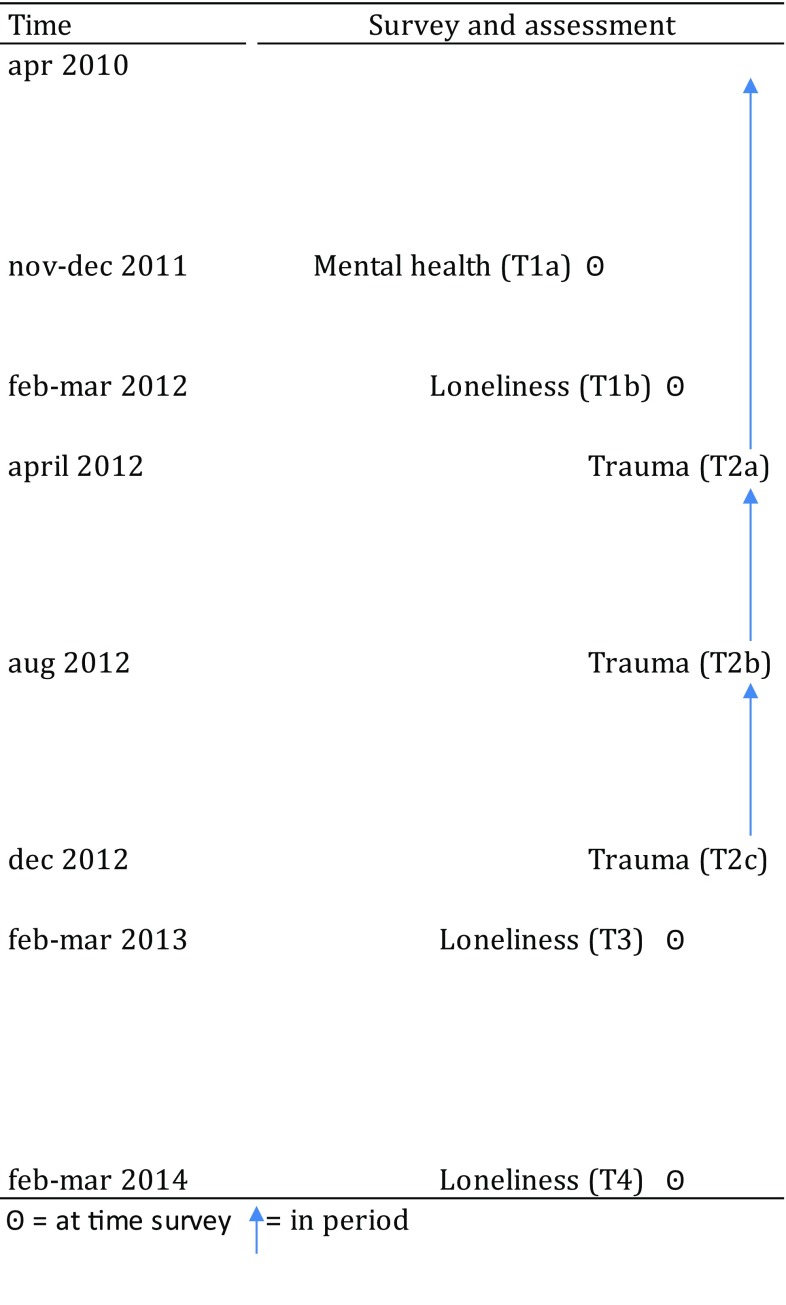
Overview surveys and nature assessments

In Fig. 1, we have presented a graphical overview of the surveys and the nature of the assessments (main topics).

### Data analysis

For the present study, several total scores were recoded. Because of the skewness loneliness scores at T1a, T3 and T4 (for instance Skewness^T1a^ = 1.494, SE Skewness = 0.058), scores were recoded (based on T1a data) into relatively high (6 thru 15; 30.5%), medium (16, 17; 30.6%) and low levels of loneliness (18; 38.9%). With respect to PTE and posttraumatic stress reactions five PTE groups were composed:

1. Respondents without PTE (not in 2 years before T2a, and not between T2a and T2c);
2. Affected respondents with very low PTSD-symptom levels (PTE between T2a and T2c, and IESplus ≤ 5);
3. Affected respondents with low PTSD-symptom levels (PTE between T2a and T2c, and 6 ≤ IEplus ≤ 17);
4. Affected respondents with medium PTSD-symptom levels (PTE between T2a and T2c, and 18 ≤ IEplus ≤ 40); and
5. Affected respondents with high PTSD-symptom levels (PTE between T2a and T2c, and IEplus ≥ 41).

The range of IESplus scores in subgroups 2–5 were chosen to obtain more or less equal numbers of respondents in each subgroup as much as possible, although this categorization is somewhat arbitrary.

The total scores of mental health problems (MHP) were divided into three levels with a more or less equal numbers of respondents: i.e. relatively low (5–8; 32.7%), medium (9–11; 34.3%), and high levels of MHP (12–hi; 33.0%) to be able to present results in the tables in a uniform way.

Because the dependent variables had three values (low, medium, high), multivariate multinomial logistic regression (MMLR) analyses were conducted with pre-event loneliness/satisfaction at T1a (2011), mental health problems at T1b (2011), PTE group membership (T2c, 2012) and as predictors. Age, gender, having a partner and education level were also added as predictors. As such, the MMLR analyses assessed to what extent the aforementioned variables predict (a) medium levels of loneliness/satisfaction at T3 (medium versus low) and (b) predict high levels of loneliness/satisfaction at T3 (high versus low).

## Results

The characteristics of the affected and non-affected respondents are presented in Table 1. According to chi-squared tests, the groups did not significantly differ on any variable of Table 1. Of the affected, 27 (9.5%) were confronted with violence/crime, 30 (10.5%) with accidents/disasters, 226 (79.3%) with the death significant other, and 6 (2.1%) with various PTE (such as suicide). In addition, 85 (29.8%) reported very low PTSD-symptom levels, 69 (24.2%) low PTSD-symptom levels, 80 (28.1%) medium PTSD-symptom levels, and 51 (17.9%) high PTSD-symptom levels.

**Table 1:** Characteristics of by potentially traumatic events affected and non-affected respondents

	Affected		Non-affected	
	*n*	*%*	*n*	*%*
Gender				
Male	143	50.2	748	49.4
Female	142	49.8	765	50.6
Age+				
34 years and younger	26	9.1	208	13.7
35–54 years	85	29.8	475	31.4
55 and older	174	61.1	830	54.9
Education				
Low	93	32.6	483	31.9
Medium	82	28.8	465	30.7
High	108	37.9	537	35.5
Unknown	2	0.7	28	1.9
Partner				
No	70	24.6	409	27.0
Yes	215	75.4	1104	73.0
Mental health problems 2011				
Low	103	36.1	485	32.1
Medium	102	35.8	514	34.0
High	80	28.1	514	34.0
Loneliness 2011				
Low	110	38.6	590	39.0
Medium	87	30.5	463	30.6
High	88	30.9	460	30.4
Loneliness 2013				
Low	132	46.3	628	41.5
Medium	72	25.3	448	29.6
High	81	28.4	437	28.9
Loneliness 2014				
Low	117	41.1	574	37.9
Medium	76	26.7	436	28.8
High	92	32.3	503	33.2

### Analyses among total study sample of affected and non-affected respondents

The main results of the MMLR analyses are presented in Table 2. The complete tables can be obtained from the first author.

**Table 2:** Prediction of loneliness among total study sample (*N* = 1798)

		Low versus medium loneliness							
		2013 (T3)^1^				2014 (T4)^2^			
	*N* ^*Total*^	*n*	%^medium^	AOR	95 CI	*n*	%^medium^	AOR	95 CI
Pre-event loneliness (2011)									
Low (Ref.)	700	642	22.3	1		602	28.6	1	
Medium	550	441	56.9	4.57	3.50–5.98***	412	52.7	2.72	2.09–3.55***
High	548	197	64.0	5.92	4.16–8.43***	189	75.1	4.31	3.02–6.15***
Pre-event mental health problems (2011)									
Low (Ref.)	588	490	32.4	1		471	36.3	1	
Medium	616	451	44.6	1.62	1.21–2.16**	417	44.1	1.32	0.99–1.75+
High	594	339	47.2	1.59	1.16–2.17**	315	49.8	1.48	1.09–2.01*
PTE groups (2012)									
No PTE (Ref.)	1513	1076	41.6	1		1010	43.2	1	
PTE very low PTSD-sl	85	68	32.4	0.53	0.31–0.93*	62	32.3	0.56	0.32–0.99*
PTE low PTSD-sl	69	50	34.0	0.67	0.35–1.27	49	46.9	1.10	0.60–2.01
PTE medium PTSD-sl	80	56	32.1	0.71	0.39–1.32	56	37.5	0.83	0.47–1.49
PTE high PTSD-sl	51	30	50.0	1.52	0.70–3.31	26	46.2	1.16	0.52–2.60

		Low versus high loneliness							
		2013 (T3)				2014 (T4)			
	*N* ^*Total*^	*n*	%^high^	AOR	95 CI	*n*	%^high^	AOR	95 CI
Pre-event loneliness (2011)									
Low (Ref.)	700	557	10.4	1		528	18.6	1	
Medium	550	299	36.5	4.74	3.28–6.83***	333	41.4	2.96	2.16–4.06***
High	548	422	83.2	35.95	24.51–52.74***	425	84.5	19.63	13.81–27.91***
Pre-event mental health problems (2011)									
Low (Ref.)	588	429	22.8	1		417	28.1	1	
Medium	616	415	39.8	1.90	1.31–2.72***	432	46.1	1.90	1.37–2.63***
High	594	434	58.8	2.69	1.88–3.85***	437	63.8	2.63	1.88–3.68***
PTE groups (2012)									
No PTE (Ref.)	1513	1065	41.0	1		1077	46.7	1	
PTE very low PTSD-sl	85	63	27.0	0.43	0.22–0.85**	65	35.4	0.61	0.33–1.12
PTE low PTSD-sl	69	52	36.5	0.73	0.36–1.51	46	43.5	0.86	0.43–1.72
PTE medium PTSD-sl	80	62	38.7	1.04	0.54–2.03	59	40.7	0.87	0.46–1.64
PTE high PTSD-sl	51	36	58.3	2.15	0.93–4.95^+^	39	64.1	2.23	1.02–4.88*

*PTSD-sl* PTSD symptom levels, *Ref*. reference group, *AOR* odds ratio adjusted for other predictors in table and age, gender, having a partner and education level at T2a, *N*^*total*^ total number of respondents in specific category, *n* number of respondents in specific category that endorsed low or medium, or low and high scores, *%medium* percentage of respondents that endorsed medium level scores of the group with low or medium level scores, *%high* percentage of respondents that endorsed high level scores of the group with low or high level scores

^+^*p* < 0.10, *p < 0.05, **p < 0.01, ***p < 0.001

^1^2013 low-medium, low–high: − 2 Log likehood = 1618.214, *χ*(30)^2^ = 763.166, *p* < 0.0001, Pseudo R-square Nagelkerke = 0.391

^2^2014 low-medium, low–high: − 2 Log likehood  = 1704.440, *χ*(30)^2^ = 579.852, *p* < 0.0001, Pseudo R-square Nagelkerke = 0.311

Table 2 shows that pre-event levels of loneliness (2011) were the strongest significant independent predictors for loneliness at T3 and T4. Analyses among those with low or medium levels of loneliness at T3 (2013) showed that the group with medium levels of loneliness at T1a (2011) had significant more often medium levels at T3 compared those with low levels of loneliness at T1a (2011). With respect to those with low or high levels of loneliness at T3 (2013), respondents with high pre-event levels of loneliness at T1a (2011) had significantly more often had high post-event levels of loneliness at T3 (2013) than those with low loneliness levels at T1a (2011). Table 2 shows the same pattern with respect to the prediction of loneliness at 2014 (T4).

Within the group of respondents with low or medium levels of loneliness at T3 (2013), affected respondents with very low PTSD-symptom levels compared to non-affected respondents significantly less often had medium levels of loneliness at T3 (2013). The same analyses among those with low or high levels of loneliness at T3 (2013) showed a similar result: affected respondents with low PTSD-symptom scores had significant less high loneliness levels at T3 (2013) than the non-affected respondents. However, there was a statistical trend (0.05 ≤ *p* < 0.10) that affected respondents with high levels of PTSD symptoms (*n* = 36) more often had high levels of loneliness than non-affected respondents. The other affected groups did not differ significantly from non-affected respondents.

A similar pattern was found predicting loneliness at T4 (2014), although affected respondents with high PTSD-symptom levels more often had high levels of loneliness than non-affected respondents at T4 (2014). The affected respondents with very low PTSD-symptom levels did not have significantly less high levels of loneliness compared to non-affected respondents. Table 2, furthermore, shows that pre-event mental health problems were independent significant predictors for post-event loneliness at both follow-ups. For instance, among those with low or high loneliness levels at T3 (2013), respondents with high pre-event levels of mental health problems more often had high levels of post-event loneliness at both follow-ups than those with low pre-event levels of mental health problems.

### Analyses among the affected respondents only

To assess differences between sub groups of affected residents, the MMLR analyses were repeated among this sub-group (*n* = 283) with the affected respondents with very low levels of PTSD-symptoms as reference group. Finding are presented in Table 3. Affected respondents with high PTSD-symptom levels more often had medium levels of loneliness at T3 (2013) than those with very low levels of PTSD-symptoms. In addition, they more often had high levels of loneliness at T3 (2013) and T4 (2014).

**Table 3:** Prediction of loneliness among affected respondents (*N* = 283)

		Low versus medium loneliness							
		2013 (T3)^1^				2014 (T4)^2^			
	*N* ^Total^	*n*	%^medium^	AOR	95% CI	*n*	%^medium^	AOR	95% CI
Pre-event loneliness (2011)									
Low (Ref.)	110	99	16.2	1		96	27.1	1	
Medium	86	67	47.8	5.69	2.58–12.51***	63	49.2	2.80	1.38–5.70**
High	87	37	64.9	11.56	4.53–29.48***	33	57.6	3.17	1.34–7.50**
Pre-event mental health problems (2011)									
Low (Ref.)	101	85	27.1	1		84	35.7	1	
Medium	102	75	41.3	2.14	0.99–4.60+	72	40.3	1.26	0.62–2.56
High	80	43	41.9	1.48	0.60–3.64	36	47.2	1.45	0.61–3.45
PTE groups (2012)									
PTE very low PTSD-sl	84	67	32.4	1		61	32.8	1	
PTE low PTSD-sl	69	50	34.0	1.15	0.47–2.81	49	46.9	1.89	0.83–4.30
PTE medium PTSD-sl	79	56	32.1	1.42	0.59–3.41	56	37.5	1.57	0.68–3.59
PTE high PTSD-sl	51	30	50.0	3.58	1.28–10.01*	26	46.2	2.16	0.79–5.91

		Low versus high loneliness							
		2013 (T3)				2014 (T4)			
	*N* ^Total^	*n*	%^high^	AOR	95% CI	*n*	%^high^	AOR	95% CI
Pre-event loneliness (2011)									
Low (Ref.)	110	94	11.7	1		84	16.7	1	
Medium	86	54	35.2	4.63	1.87–11.49***	55	41.8	3.44	1.48–8.01**
High	87	63	79.4	28.94	11.11–74.42***	68	79.4	16.04	6.60–39.01***
Pre-event mental health problems (2011)									
Low (Ref.)	101	78	20.5	1		71	23.9	1	
Medium	102	71	38.0	2.61	1.09–6.21*	73	41.1	2.01	0.91–4.81+
High	80	62	59.7	3.78	1.50–9.51**	63	69.8	4.34	1.76–10.72***
PTE groups (2012)									
PTE very low PTSD-sl	84	62	27.4	1		64	35.9	1	
PTE low PTSD-sl	69	52	36.5	1.63	0.62–4.34	46	43.5	1.44	0.57–3.61
PTE medium PTSD-sl	79	61	37.7	2.25	0.89–5.77+	58	39.7	1.42	0.58–3.45
PTE high PTSD-sl	51	36	58.3	5.24	1.76–15.58**	39	64.1	3.13	1.12–8.69*

The total number of affected respondents is lower than in Table 1 (285 versus 283) because for two respondents the education was unknown and, therefore, were omitted from the analyses

*PTSD-sl* PTSD symptom levels, *Ref*. reference group, *AOR* odds ratio adjusted for other predictors in table and age, gender, having a partner and education level at T2a,* N*^*total*^ total number of respondents in specific category, *n* number of respondents in specific category that endorsed low or medium, or low and high scores, *%medium* percentage of respondents that endorsed medium level scores of the group with low or medium level scores, *%high* percentage of respondents that endorsed high level scores of the group with low or high level scores

+*p* < 0.10, *p <0.05, **p < 0.01, ***p < 0.001

^1^2013 low-medium, low-high: −2 Log likehood = 428.413, *χ*(26)^2^ = 127.000, *P* < .0001, Pseudo R-square Nagelkerke = .411

^2^2014 low-medium, low-high: −2 Log likehood = 452.678, *χ*(26)^2^ = 112.724, *P* < .0001, Pseudo R-square Nagelkerke = .371

Additional and identical analyses with being affected (PTE is yes or no) as predictor instead of the five categories based on PTE and PTSD-symptoms showed that PTE did not significantly (*p* < 0.05) and independently predict post-event loneliness at both follow-ups (data not shown).

## Discussion

Aim of the present study was to examine to what extent adults affected by potentially traumatic events (PTE) with different levels of posttraumatic stress symptom are more at risk for post-event loneliness at different post-event stages than non-affected adults, while taking pre-event loneliness and pre-event mental health problems into account.

### Main findings

Remarkably, we found both negative as well as positive effects of PTE and PTSD symptoms on post-event loneliness.

The results of the multivariate multinomial logistic regression analyses showed that affected respondents with high levels of PTSD-symptoms were more at risk for relatively high levels of loneliness at the second follow-up than non-affected respondents. There was a statistical trend that they were also more at risk for high levels of loneliness at the first survey than non-affected. However, with respect to the prediction of high levels of loneliness no significant differences were found between non-affected at the one side and affected respondents with low and medium levels of PTSD-symptoms on the other side. In addition, respondents with different levels of posttraumatic stress symptoms were not more at risk for medium levels of loneliness than non-affected respondents at both follow-ups. However, in line with previous research on loneliness and social support [40, 51, 52], pre-event loneliness appeared to be a strong predictor of post-event loneliness at both follow-ups.

Pre-event mental health problems were also clearly associated with loneliness at follow-ups: existing mental health problems were a risk factor for loneliness about one and two years later. The analyses among the affected respondents revealed that, in contrast to, for example, the study of Solomon et al. [38], affected adults with very low PTSD-symptom levels were less at risk for high levels of loneliness at both follow-ups than affected respondents with high levels of PTSD-symptoms. Moreover, the affected respondents with very low levels of PTSD-symptoms were *less* at risk for medium and high levels of loneliness than non-affected respondents at the first follow-up and less at risk for medium levels of loneliness at the second follow-up.

### Social causation

How can these contrasting negative and positive findings be explained? Kaniasty and Norris [53] showed two causal pathways in which social support and PTSD-symptoms could influence each other, specifically social causation and the social selection. In social causation, the lack of social support will increase the chances for the development of PTSD-symptoms at later stages, but in social selection higher PTSD-symptom levels will decrease social support at later stages. The outcomes of the comparisons between affected respondents with very low levels of PTSD-symptoms and affected residents with high levels of PTSD-symptoms are in line with the social selection perspective: higher PTSD-symptom levels were predictive of higher loneliness levels [54]. The positive association between pre-event mental health problems and higher post-event perceived loneliness also points to the presence of social selection.

Our results are also in line with the cognitive model of Ehlers and Clark [20]. Victims suffering from PTSD symptoms may appraise their situation as very negative and see their reactions to the event as indicators that they have changed for the worst, and not that their reaction are part of a normal process. This may lead to social withdrawal and therefore higher levels of loneliness. An important element in this process is the response of family, friends and colleagues. They may find it difficult to respond because of the PTE persons experienced and especially their posttraumatic stress symptoms, and therefore avoid talking about experiences and problems. This is also expressed by associations between PTSD and, among others, social anxiety and family problems [55].

Our finding that affected residents with very low PTSD-symptom levels were even *less* lonely that non-affected residents may also fit in the cognitive model Ehlers and Clark [20]. A possible explanation in this perspective is that victims experienced a potential traumatic event but because of the absence of mild or severe PTSD-symptoms these problems no longer form a barrier making it more convenient for family and friends to respond, talk about it, and provide recognition resulting in a decrease in loneliness. Future longitudinal research is warranted to confirm (or reject) our findings and explanation that PTE in the absence of mild-severe PTSD-symptoms decrease loneliness. However, these results and the social selection perspective do show support that PTSD interventions directed towards more severe PTSD symptomology could benefit from addressing social functioning problems.

### Limitations and strengths

Several characteristics and limitations need to be discussed. The prospective comparative design to assess the effects of PTE and PTSD-symptoms on post-event loneliness can be considered a major strength of our study. We excluded respondents that were affected by PTE in the past 2 years, to ascertain as much as possible that findings were not biased by previous events and their possible effects on loneliness. Comparisons between affected and non-affected showed that pre-event loneliness levels as well as pre-event mental health problems were similar between both groups (as well as demographic characteristics). To the best of our knowledge, to date no study assessed the effects of PTE and PTSD-symptoms using such a rigorous study design. We examined the effects of recent potentially traumatic events. We did not examine the effects of events experiences during childhood, such as child sexual abuse and childhood physical abuse [56].

We used well-validated questionnaires to examine mental health problems, loneliness, and posttraumatic stress symptoms. We did not conduct clinical interviews to assess posttraumatic stress disorder that would have enriched our study. In addition, although social support and loneliness are related concepts, we have no data on either pre- or post-event social support. We assessed the predictive values of PTE and PTSD-symptoms for loneliness at two follow-ups with a 1-year time interval, and focused on the effects of relatively recent PTE. Future research should focus on the long-term effects of PTE and PTSD symptoms on loneliness, and the possible effects of re-victimization and child abuse on loneliness.

There was a gap of about 3 months between the measurement of (pre-event) mental health (T1a) and (pre-event) loneliness (T1b). However, since previous research has shown that these mental health problems are rather stable [57], we do not expect that this gap influenced our findings. There was also a gap of about 2–3 months between the PTSD-symptomatology (T2c) and the second or post-event loneliness assessment (T3). The last loneliness assessment (T4) took place 1 year later. We have no information about confrontations with PTE after the last PTE examination (December 2012) and these events and/or related PTSD-symptomatology may have weakened associations, especially with respect to last follow-up assessment of loneliness (T4).

Another possible explanation of the absence of large differences between non-affected and affected people with relatively high levels of PTSD symptoms is an underreporting of loneliness among these affected respondents because of the negative (emotional) connotation of loneliness. However, to prevent this problem the De Jong Gierveld Loneliness Scale was constructed without using the word loneliness, although items are directly related to the conceptual ideas of loneliness, [25]. For this reason, we consider this explanation not very plausible.

Finally, a previous study [57], using the LISS panel including the surveys on trauma, has shown that the non-response did not or hardly affect the mental health scores: “Those participating in 1, 2, 3, 4, 5, 6 or 7 surveys did not differ on three of the four health variables. The significant differences in mental health between those participating in 7 surveys and other groups were (very) small according to Cohens D and did not show a clear pattern (p. 469)”.

### Implications for further research

It was outside the aim of the present study to assess differences between pre-event situational versus pre-event chronic loneliness, besides the fact that numbers of affected respondents in our study were too low to analyze possible differences. However, in future research this distinction may be relevant given the results of for instance the longitudinal study of Shiovitz-Ezra and Ayalon [58]. Both situationally, loneliness and chronic loneliness were associated with a greater risk for all-cause mortality when controlled for demographics and health confounders, but chronic loneliness was a stronger predictor.

The study of Fokkema et al. [59] revealed differences in loneliness levels between older adults of 14 EU countries, while controlling for demographics, health, social embeddedness and support exchange. In addition, Koenen et al. [60] showed that, based on a comparison of 26 population WHO World Mental Health Surveys, lifetime and 12-month prevalence of PTSD differed between countries. Therefore, future prospective research is needed among population-based samples in other countries to assess the influence of country or context differences and strengths of associations between potential traumatic events, PTSD symptomatology and loneliness.

The finding that victims with high levels of PTSD-symptoms are more at risk for high levels of loneliness than non-affected adults and PTE affected adults with low levels of PTSD raises the question how this risk can be prevented or reduced. Two recent reviews, however, not specifically aimed at trauma, are of relevance here. The review of Mann et al. [61] on interventions to reduce loneliness demonstrated that, although ‘changing cognitions’ had the most promising emerging evidence according to the authors, no interventions had robust evidence. The review of Webber and Fendt-Newlin [62] on the effects of social participation interventions for people, including those provided by mental health services, with mental health problems concluded “A wide range of approaches are used to help improve the social participation of people with mental health problems, though evidence of their effectiveness is minima (p.)”. These results indicate that further research on interventions is needed to address this problem in general and specifically with respect to potential traumatic events.

### Finally

In either way, the paramount role of pre-event loneliness suggests that post-event social problems might be rooted in a wider context of social dysfunction of the affected individual, rather than to be directly induced by the traumatic experience. In either way, the paramount role of pre-event loneliness suggests that for the early identification of those at risk for post-event social problems, the assessment of pre-event loneliness should be included.

## Footnotes

1 See http://home.fsw.vu.nl/tg.van.tilburg/manual_loneliness_scale_1999.html#short_version.
